# The General Practitioner and Sanatorium Benefit

**Published:** 1912-08-03

**Authors:** 


### THE GENERAL PRACTITIONER AND SANATORIUM BENEFIT.

On February 22 of this year the Treasury appointed the Tuberculosis Committee to consider the general policy in respect to the problem of tuberculosis in the United Kingdom in its preventive, curative, and other aspects, with a view to guiding the Government and local bodies in making and aiding provision for the treatment of tuberculosis in sanatoria and other institutions or otherwise, such treatment to commence on July 15. With admirable promptness the Committee published an interim report on April 30, and on the basis of this report sanatorium benefit will come into operation; though in a letter dated June 27 the Committee state that some time must necessarily elapse before schemes on the lines suggested can be brought into operation.

In conjunction with their medical officers of health, the county and borough councils have taken steps to arrange the administration of sanatorium benefit, and the medical profession and medical officers of health will co-operate in administering such benefit "provided it is carried on in accordance with the wishes of the profession." We wish to call attention to certain points in the interim report which must be clearly defined. We think that the right and suitability of the general practitioner to administer sanatorium benefit should be acknowledged, though he might work under a whole-time medical officer of special experience. In order to avoid the misuse of voluntary hospitals and other charities in giving gratuitous medical attendance to State-aided patients, if such institutions are used at all, the accounts of such treatment must be kept entirely separate from the ordinary accounts of the institution, and adequate payment must be made to practitioners giving the treatment. Also the tuberculosis scheme must be confined to insured persons. Should any wider scheme be promulgated to embrace the "whole community" payment must be ensured according to the social position of the tuberculous person. If a person diagnosed to be suffering from tuberculous disease is unable for want of sufficient funds or accommodation to obtain sanatorium benefit, such person must only obtain ordinary medical benefit as an extra.

A memorandum issued in July by the National Health Insurance Commissioners (England) after conference with the Local Government Board advises Insurance Committees that, though they may extend treatment to the dependants of insured persons, it is desirable to confine the benefit to insured persons until the financial liabilities of the Committee can be more adequately estimated; also that in administering the benefit Insurance Committees are not empowered by the Act to provide institutions—their duty is to make arrangements with suitable persons or local authorities for this purpose. It advises that the Insurance Committees shall at an early date place themselves in communication with the county or borough councils with a view to the medical officer of health attending a meeting of the Committee. Where a tuberculosis officer has not been appointed, and the agencies available are very limited, the medical officer of health may be appointed to act temporarily as the expert adviser of the Committee in dealing with individual applications for sanatorium benefit. Where a patient is waiting for admission to a hospital or sanatorium, home treatment by a private practitioner or a dispensary is recommended, the Committee bearing the expense. In connection with the memorandum the Local Government Board have issued a circular letter to the county and borough councils expressing the hope that the services of the medical officers of health may be available for administering sanatorium benefit at the earliest practicable date. Both the Commissioners and the Local Government Board concur in thinking that the home treatment of tuberculous persons should, as far as possible, be carried out by general practitioners acting under an approved adviser, the hygienic conditions being suitable.

